# Longitudinal MRI Study on the Natural History of Carotid Artery Plaques in Symptomatic Patients

**DOI:** 10.1371/journal.pone.0042472

**Published:** 2012-07-31

**Authors:** Robert M. Kwee, Martine T. B. Truijman, Robert J. van Oostenbrugge, Werner H. Mess, Martin H. Prins, Cees L. Franke, Arthur G. G. C. Korten, Joachim E. Wildberger, M. Eline Kooi

**Affiliations:** 1 Department of Radiology, Maastricht University Medical Center, Maastricht, The Netherlands; 2 Cardiovascular Research Institute Maastricht, Maastricht University Medical Center, Maastricht, The Netherlands; 3 Department of Clinical Neurophysiology, Maastricht University Medical Center, Maastricht, The Netherlands; 4 Department of Neurology, Maastricht University Medical Center, Maastricht, The Netherlands; 5 Department of Clinical Epidemiology and Medical Technology Assessment, Maastricht University Medical Center, Maastricht, The Netherlands; 6 Department of Neurology, Atrium Medical Center Parkstad, Heerlen, The Netherlands; 7 Department of Neurology, Laurentius Hospital, Roermond, The Netherlands; Innsbruck Medical University, Austria

## Abstract

**Purpose:**

To investigate the natural history of carotid atherosclerosis in patients who experienced a TIA or ischemic stroke.

**Patients and Methods:**

Ninety-two TIA/stroke patients (57 men, mean age 67.7±9.8 years) with ipsilateral <70% carotid stenosis underwent multisequence MRI of the plaque ipsilateral to the symptomatic side at baseline and after one year. For each plaque, several parameters were assessed at both time points.

**Results:**

Carotid lumen, wall and total vessel ( = carotid lumen and wall) volume did not significantly change. Forty-four patients had a plaque with a lipid-rich necrotic core (LRNC) at baseline, of which 34 also had a LRNC after one year. In three patients a LRNC appeared after one year. Thirty patients had a plaque with a thin and/or ruptured fibrous cap (FC) at both time points. In seven patients, FC status changed from thin and/or ruptured into thick and intact. In three patients, FC status changed from thick and intact into thin and/or ruptured. Twenty patients had intraplaque hemorrhage (IPH) at both time points. In four patients, IPH disappeared, whereas in three patients, new IPH appeared at follow-up.

**Conclusion:**

In TIA/stroke patients, carotid plaque morphology does not significantly change over a one-year period. IPH and FC status change in a minority of patients.

## Introduction

Carotid atherosclerosis is an important cause of ischemic stroke. Plaque disruption with superimposed thrombus formation and subsequent embolization are thought to be the causative mechanisms [1], [2]. Carotid endarterectomy has shown to be beneficial in symptomatic patients (i.e. those who experienced a TIA or ischemic stroke) with >70% stenosis [3]. In patients with 30–69% stenosis, the benefit is less clear [3]. Little has been published on the natural history of carotid plaques in symptomatic patients [4]. Knowledge on the natural history may help improving understanding of atherosclerosis and may enable management of modifiable risk factors. MRI is an excellent tool to monitor vessel wall morphology and key plaque components, with good accuracy and reproducibility [5], [6]. Therefore, the purpose of the present study was to investigate the natural course of carotid plaques in symptomatic patients, using MRI. Results of the first 40 patients have been reported previously in a pilot study [4]. The present study was expanded with another 52 patients.

## Patients and Methods

### Patients

Patients who were diagnosed by a neurologist as having recent (<3 months) TIA or minor stroke and an ipsilateral carotid plaque causing 30–69% stenosis, as measured at ultrasonography (US), were eligible for inclusion. 30–69% carotid stenosis was defined as a luminal diameter reduction of at least 30% on transverse B-mode US images (according to ECST criteria) [7] and a maximum peak systolic velocity of 230 cm/s at the site of maximal luminal narrowing [8]. Exclusion criteria were: atrial fibrillation, claustrophobia, and other standard contraindications for MRI [9], and a renal clearance <30 ml/min/1.73 m^2^. This study was approved by the institutional review board of our hospital. All patients gave written informed consent.

### MRI protocol and image review

The MRI protocol and method of image review has been described in detail previously [4], [10]. MRI examinations were performed on a 1.5-T scanner (Intera 11.1.4.3, Philips Healthcare, Best, the Netherlands). A dedicated 47 mm-diameter surface coil (Philips Healthcare) was fixed to the skin at the level of the carotid bifurcation at the symptomatic side. The plaque was imaged within nine transverse slices. If the plaque was located symmetrically around the bifurcation (which was mostly the case), the analyzed slice package was also located symmetrically around the bifurcation. In other cases, the analyzed slice package was a little bit asymmetrical around the bifurcation (i.e. more slices below than above the bifurcation, or vice versa). Five MR pulse sequences were acquired: (1) 3D T1-weighted TFE: TR/TI/TE 10.3/900/4.4 msec, flip angle 15°, NSA 6, inversion prepulse, shot interval time 3000 msec, TFE factor 163, profile order: linear, slice thickness 3.0 mm; (2) 3D TOF: TR/TE 23/3.9 msec, flip angle 25°, NSA 4, slice thickness 3.0 mm; (3) Multislice T2-weighted TSE: TR/TE 2 heartbeats/50 msec, NSA 2, echo train length 8, profile order: linear, slice thickness/gap 2.5/0.5 mm; (4/5) Pre- and post-contrast 2D T1-weighted TSE (double inversion-recovery): TR/TI/TE 1 heartbeat/heart rate dependent/18 msec, NSA 2, echo train length 9, profile order: linear, slice thickness/gap 2.5/0.5 mm. The post-contrast T1-weighted TSE sequence was obtained 7–8 minutes after injection of 0.1 mmol/kg body weight of gadopentetate dimeglumine (Magnevist, Bayer Schering Pharma AG, Berlin, Germany). TI was adjusted according to heart rate and postcontrast T1 relaxation time of blood. For all sequences the FOV was 100×80 mm, with a matrix size of 256×205 (in-plane resolution, 0.39×0.39 mm), except for the T1-weighted TFE sequence (FOV 100×80 mm, matrix size 256×163; in-plane resolution 0.39×0.49 mm).

**Figure 1:**
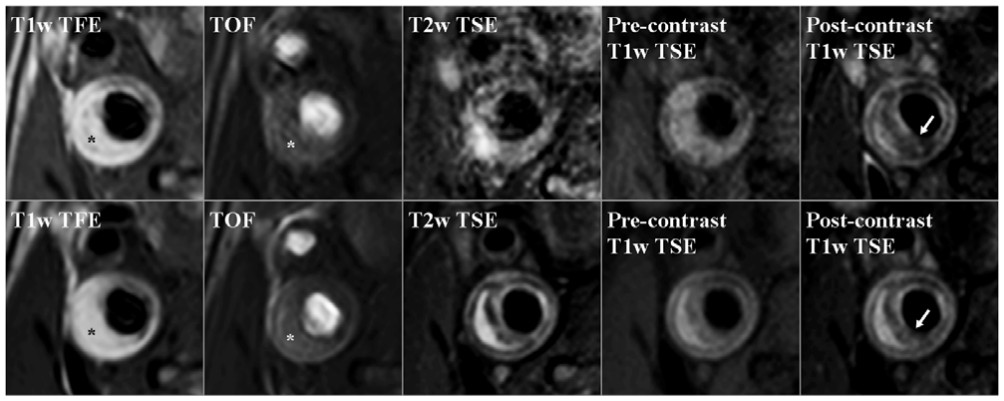
Multisequence MR images of a plaque in the internal carotid artery obtained at baseline (upper row) and after one year (bottom row). The TOF and T2w TSE images at baseline are slightly blurred due to patient motion. There is no obvious change of plaque morphology. There is a plaque with a LRNC and IPH (asterisks) at baseline and at 1-year follow-up. There is a disruption of the FC at both time points (arrows).

MR images were evaluated by one investigator with 3 years of experience in plaque analysis by MRI (R.M.K.), blinded to all patient data and the time point at which the MRI examination was performed. MR images were evaluated using dedicated vessel wall analysis software (VesselMASS, Department of Radiology, Leiden University Medical Center, The Netherlands) [4], [10]. TOF images were used to identify the vessel lumen (bright signal) and to distinguish it from potential juxtaluminal calcifications (hypointense signal). T1w TFE, T2w TSE, pre- and post-contrast T1w TSE images were used to identify the vessel wall. Regions of interest (ROIs) were drawn around the lumen area and the vessel wall on all slices. The software calculated carotid lumen volume, wall volume, and total vessel volume ( = carotid lumen volume+wall volume). When there is carotid plaque signal hyperintensity on the T1w TFE and/or TOF images, it indicates the presence of intraplaque hemorrhage (IPH) within a lipid-rich necrotic core (LRNC) [11], [12]. When there is an area in the bulk of the plaque with no or slight contrast enhancement on post-contrast T1w TSE images, and no high signal on the T1w TFE and/or TOF images, it indicates the presence of a LRNC without IPH [11]–[13]. Fibrous cap (FC) status was also assessed: when a disrupted or discontinuous high signal between LRNC and the lumen was identified on at least one of the post-contrast T1w TSE images, FC status was classified as “thin and/or ruptured”. Alternatively, it was classified as “intact and thick” [6]. Figure 1 gives an example of multisequence MR plaque imaging performed at baseline and after one year.

### Statistical analysis

Statistical analysis was performed using SPSS version 18.0 (SPSS Inc, Chicago, USA). Differences between baseline and after one-year follow-up were assessed by paired-samples T-tests. Change in plaque morphology was defined as a significant change in carotid lumen, wall, or total vessel volume. Differences in change in plaque morphology between patients who experienced a TIA and patients who experienced a (minor) stroke were assessed by independent-samples T-tests. All levels of significance were set at 0.05. Changes in the presence of complicated plaque characteristics (LRNC, IPH, and FC status) were described.

## Results

One hundred and twenty-six patients were included. In 3 patients, (baseline) MR images were of insufficient quality so that carotid plaque assessment could not be performed. In two patients, the longitudinal coverage of repeated MRI did not match those of the baseline MRI, so that plaque measurements at both time points could not be fairly compared. In one patient, carotid stenosis grade appeared to be >70% at baseline MRI and carotid endarterectomy was performed; this patient therefore did not undergo repeated MRI. Another 28 patients also did not undergo repeated MRI of the carotid plaque after one year, because of withdrawal (n = 24), death due to myocardial infarction (n = 1), pacemaker implantation (n = 1), recurrent stroke and subsequent carotid endarterectomy (n = 1), and death due to recurrent stroke (n = 1). Eventually, in 92 patients (57 men, mean age 67.7±9.8 years) baseline and follow-up MRI scans of the plaque were compared. There were 45 patients who experienced a TIA and 47 patients who experienced a (minor) stroke. Mean time interval between last symptoms and baseline MRI examination of these patients was 33.1±18.5 days. According to clinical guidelines, all patients were prescribed aspirin (100 mg once a day) and dipyridamole (200 mg twice a day) for secondary stroke prevention. Seventy-nine patients used statins (atorvastatin 10–40 mg, pravastatin 20–40 mg, rosuvastatin 5–20 mg, or simvastatin 10–40 mg once a day) during the entire follow-up period. Eighteen patients continued smoking during the follow-up period.

For the whole group, carotid lumen volume, wall volume, and total vessel volume did not significantly change (Table 1). There also was no significant difference in change of aforementioned measurements between patients who experienced a TIA and patients who experienced a (minor) stroke (*P*-values = 0.54, 0.84, and 0.45, respectively). Forty-four patients had a plaque with a LRNC at baseline, of which 34 also had a LRNC after one year. In three patients, a LRNC appeared after one year. Thirty patients had a plaque with a thin and/or ruptured FC at both time points. In seven patients, FC status changed from thin and/or ruptured into thick and intact. In three patients, FC status changed from thick and intact into thin and/or ruptured. Twenty patients had IPH both at baseline and at follow-up. In four patients, IPH disappeared, whereas in three patients, new IPH appeared at follow-up.

**Table 1:** Volumetric measurements (mm^3^) at baseline and after one year.

	Baseline	One year	Δ (%)	*P*-value
**Carotid lumen volume**	939±275	960±299	2.5±12.8	0.10
**Wall volume**	969±304	974±313	1.4±15.3	0.75
**Total vessel volume**	1908±493	1933±491	1.9±8.9	0.16

## Discussion

The results of the present study show that plaque morphology in TIA/stroke patients with 30–69% carotid stenosis does not significantly change over a one-year period. The presence of a LRNC, IPH and FC status (vulnerable plaque characteristics) change in a minority of patients.

Plaques of symptomatic patients are probably of a different entity compared to plaques in asymptomatic patients. Despite equal stenosis grade, symptomatic patients have a higher risk of (recurrent) stroke compared to patients with asymptomatic carotid stenosis, as was shown by large randomized controlled trials [14], [15]. In addition, it has been shown that symptomatic and asymptomatic plaques have a different prevalence of certain plaque features at MRI [16], [17]. Previous studies by Saam et al. [18] and Boussel et al. [19], investigating the natural course of carotid plaques in 68 and 160 (mainly) asymptomatic patients, found that mean wall area and wall volume increased by as little as 2.2% and 2.31% per year, respectively. Another study in 67 asymptomatic patients with 16–49% carotid stenosis, by Underhill et al. [20], found no significant change in the percentage of wall volume per year, which is in accordance to the results of the present study. All aforementioned studies [18]–[20] found a reduced rate of plaque growth in patients who used statins. In accordance, a recent randomized trial showed that intense use of statins causes regression in overall plaque burden in patients with asymptomatic carotid atherosclerosis, which was already observed after one year of treatment [21]. Underhill at al. [20] found that the presence of IPH was associated with plaque growth. Because we did not find a significant change in plaque morphology, we also did not investigate any possible effect of cardiovascular risk factors, statin use, or the presence of certain plaque characteristics. Whether statin use and the presence of IPH are associated with a change in plaque morphology in symptomatic patients remains to be investigated.

Our study had several limitations. First, there was a large variation in time interval between last symptoms and baseline MRI examination (33.1±18.5 days), caused by study referral delay, patient and scanner availability. To our knowledge, there is not much known on how carotid plaques change in the initial high-risk phase [22] after the event, and it is unclear how this variation might have affected our results. Second, the size of the current study was relatively small. Our previously reported study, comprising the first 40 patients of the current one, was a pilot study, and the effect we found in that study was probably false positive. Although we more than doubled our previously reported series [4], we did not detect a significant change in plaque morphology in the present study. From a post-hoc power analysis (80% power and a 2-sided type 1 error of 0.05), based on the current observations with a difference that is 0.1–0.2 of its SD, we learned that 200 to 800 patients with repeated measurements are required to detect a significant change in one year. Third, longer follow-up might also have detected more change in plaque morphology. Another study limitation was the relatively large number of patients (22%) who withdrew and did not undergo MRI of the plaque after one year. Notably, two patients did not undergo imaging after one year due to recurrent stroke. Fourth, patients were not randomized in receiving statin therapy, 37 patients already used statins before the initial event, and the patients who used statins used different types and dosages of statins. Therefore, any potential effect of statin use on carotid plaque morphology and composition in symptomatic patients should be further investigated. Last, reproducibility for the assessment of volumes of individual plaque components (such as volume of LRNC and calcifications) was insufficient (coefficients of variation≥30%) and these parameters were therefore not included in the present follow-up study.

In conclusion, we found that carotid plaque morphology of symptomatic patients does not significantly change over a one-year period. Whether these plaques are dormant and whether patients with these kind of plaques do not have to be followed should be determined by larger studies with longer patient follow-up.
